# A Randomized Trial on the Effect of Razavi's Dietary Pattern on the Components of Metabolic Syndrome

**DOI:** 10.5812/ircmj.14601

**Published:** 2014-03-05

**Authors:** Seied Saeid Esmaeili, Faramarz Fallahi, Mohammad Gholami Fesharaki, Gholamreza Noormohammadi

**Affiliations:** 1 Department of Iranian Traditional Medicine, Faculty of Medicine, Shahed University, Tehran, IR Iran; 2 Department of Cardiology, Mostafa Khomeyni Hospital, Faculty of Medicine, Shahed University, Tehran, IR Iran; 3 Department of Biostatistics, Faculty of Medical Sciences, Tarbiat Modares University, Tehran, IR Iran; 4 Department of Maaref, Tehran University, Tehran, IR Iran

**Keywords:** Metabolic Syndrome X, Obesity, Diet therapy

## Abstract

**Background:**

Metabolic syndrome can cause cardiovascular disease and diabetes in the affected subjects. With 20 to 30% prevalence rate among the adult population of most countries, it is considered a pandemic problem. The guidelines currently available on the management of the specific components of metabolic syndrome highlight some lifestyle changes such as enhanced physical activity and weight reduction. Adherence to Mediterranean-style diet has been shown to be associated with lower risk of metabolic syndrome in some clinical studies.

**Objectives:**

The aim of this study was the evaluation of the effect Razavi dietary pattern, on metabolic syndrome. This is the first study performed to address this issue.

**Patients and Methods:**

Seventy five eligible subjects with metabolic syndrome were recruited into a single-blind randomized controlled clinical trial to determine the effect of Razavi diet on metabolic syndrome. Intervention was carried out by educating the Razavi diet in the experimental group while giving no dietary recommendations to the control group. The level of physical activity was similar between the two groups. Features of the metabolic syndrome as defined by the criteria of the Iranian National Committee of Obesity were assessed after two months.

**Results:**

The net reduction in the waist circumference (-2.85), weight (-1.44) and BMI (-0.58) in test group was significantly (P < 0.001) higher than the control. Decreases in systolic and diastolic blood pressure, fasting blood sugar and triglycerides were observed but were not statistically significant.

**Conclusions:**

The results suggest that Razavi diet can improve some components of metabolic syndrome leading to reduced risk of cardiovascular disease and diabetes.

## 1. Background

Metabolic syndrome (MS) has recently become a focus of attention (1). MS is defined as having any three of the centrally distributed obesity, reduced HDL (high-density lipoprotein), hypertriglyceridemia, high blood pressure (BP), and hyperglycemia (2). Matabolic syndrome is associated with two fold increase in the risk of cardiovascular disease compared to those without the syndrome. It also raises the risk of type II diabetes by about 5-folds. The available evidence indicates that 20-30% of the adult population in most countries are affected by the MS which is called the metabolic syndrome pandemic (3). In men, the syndrome increases cardiovascular disease (CVD) and all-cause mortality even in the absence of baseline CVD and diabetes (4). An important approach for the reduction of the CVD burden could be the diagnosis, prevention, and treatment of the causal risk factors of the metabolic syndrome in the general population (5). MS increases risk of cancers particularly cancer of prostate (6) and colon (7). The prevalence of this syndrome among Iranian adults is 32.1% by the IDF definition and 33.2% by the ATPIII definition (8) and 10.1% among Iranian adolescents (9). These prevalence rates are among the highest in the world (10).

The guidelines currently available on the management of the specific components of MS emphasize the lifestyle changes such as enhanced physical activity and weight loss as the first step in treatment (11). Higher risks of insulin resistance and also MS are reported to be associated with the western style diet whereas no significant correlation is reported to exist between the traditional style diet and these conditions (12). Adherence to the Mediterranean diet is shown to be associated with reduced risk of metabolic syndrome in some clinical trials (13). We could not find any research report examining the effect of dietary patterns advised by the Iranian traditional medicine or Islamic medicine on metabolic syndrome.

## 2. Objectives

In this study, the effect of Razavi style-diet on MS (14) was assessed. This pattern is driven from the text known as "Resaleh Zahabieh" meaning “Golden Letter” that belongs to Ali Ibn Musa (Imam Reza), the 8th Imam of Shiite sect of Islam. This text was written about 779-782 while Imam Reza lived in Marv, the Abbasid’s capital city during the al-Ma'mun’s governance (813-833). This text was different from many books in this field in certain ways. Thus, al-Ma'mun commanded some scribes to write the text with gold and therefore it is widely known as "Resaleh Zahabieh" which means golden letter. According to the Resaleh, one of the most important ways to prevent and treat illnesses is lifestyle interventions such as corrections in the nutritional pattern, sleep and physical activity. This nutritional pattern suggests appropriate quantity, quality, feeding times and suitable diet for each season and each month. But it does not emphasize the current advice such as restriction of saturated fat and increase in consumption of high fiber/low-glycemic-index foods (15). Further clarification of this nutritional pattern will be given in the methods section. This is the first research report on the impact of this pattern on MS.

## 3. Patients and Methods

Of 110 individuals with serum lipid abnormalities, hyperglycemia, abnormally high weight or high BP referred to our medical center in Tehran, Iran, 75 participants were selected as having metabolic syndrome criteria. An informed consent was obtained from each subject. As a screening tool, personal health and medical history questionnaire was completed by each contributor. The age range of participants was 20-70 years old. Based on the Iranian National Committee of Obesity (INCO) criteria (10), MS was diagnosed as having at least three of the following criteria (Table 1). Patients with renal failure or liver insufficiency, cancer, acute cardiovascular syndrome, acute infectious diseases and uncontrolled diabetes were excluded. Ethics approval was from ethics committee of Shahed University, Tehran-Iran. This RCT has registered in the Iranian Registry of Clinical Trials (IRCT) site and registration ID in IRCT is IRCT2012111111431N1.

**Table 1.** Criteria for Clinical Diagnosis of Metabolic Syndrome in Iranian Adults

Criteria	Results
**Elevated waist circumference, cm**	95
**Elevated triglycerides, 17 mmol/L**	150
	drug treatment for elevated triglycerides
**Reduced HDL-C, 1.0 mmol/L**	40 in male
	50 in female
	drug treatment for reduced HDL-C
**Elevated blood pressure, mm Hg**	Systolic 130 and/or diastolic 85
	antihypertensive drug treatment in
	a patient with a history of hypertension
**Elevated fasting glucose, mg/dL**	100
	drug treatment of elevated glucose

### 3.1. Sample Size

The following formula was used to estimate the sample size (Equation 1): 

Equation 1. $$n=\frac{{\left(Z_{\alpha }+Z_{\beta }\right)}^{2}}{\delta^{2}}$$

Where (Equation 2): 

Equation 2. $$\delta^{2}=\frac{{(\mu_{1\mathrm{diff}}-\mu_{2\mathrm{diff}})}^{2}}{\sigma_{\mathrm{diff}1}^{2}+\sigma_{2\mathrm{diff}}^{2}}$$

α: Type I error, β:Type II error and Z_p_: is quintile of standard normal distribution with probability p.

Considering α= 0.05 (Z_0.05_= 1.63), β=0.1 (Z_0.1_ =1.28) and moderate size index effect (δ = 0.05) the sample size in each group was computed as follows (Equation 3):

Equation 3. $$n=\frac{{\left(Z_{\frac{\alpha }{2}}+Z_{\beta }\right)}^{2}}{\delta^{2}}=\frac{8.64}{0.5^{2}}=\frac{8.64}{0.25}=34.56=35$$

Assuming a drop-out rate of 10%, the total sample size was estimated to be 38 subjects in each group, so in this study a total of 76 subjects were enrolled. This study was a randomized single-blind clinical trial. The participants were randomly allocated into two groups: 38 patients receiving the Razavi dietary pattern and 37 patients in the control group. None of the subjects or laboratory staff were aware of the group assignments. The diet instructor was aware of the group assignment.

### 3.2. Control Diet

No diet prescription other than “eat as usual” was given to the control group.

### 3.3. Razavi Nutritional Pattern

In intervention group, volunteer subjects took nutrition education in a 2-hour session hold for the study group. An educational brochure was delivered and a self-assessment form (nutrition diary) was completed daily. Meetings were held to follow up, provide answers to participants' questions and resolve their issues every two weeks. According to this nutritional pattern, participants were advised to act in accordance with the followings:

A. Quantity: 1. Moderate diet: Eat with real appetite and avoid eating when you have little appetite.

2. Meals decussate: a complete meal then a potluck meal and so on.

B. Quality: 1. Balance and harmony with the person's temperament and suitability for the stomach status.

2. Foods with warm nature in winter and cold in summer and moderate in other seasons are recommended.

C. Observe a specific sequence in eating: eating should start with snacks and soft foods followed by heavy or solid meal.

D. Moderate drinking and no water or beverages with food.

### 3.4. Study Procedures

Each patient was followed up every week during the two months of intervention. Participants did not change their habitual physical activity levels during the course of the study. They were also advised to take their routine medications.

### 3.5. Measurements

The subjects’ weight measurement was performed using digital scales while they were wearing minimal clothing. Subjects' height were measured with a wall mounted stadiometer in upright position with the shoulders in normal state and shoes removed. An un-stretched tape meter was used to measure the waist circumference at the umbilicus level (midway between the lowest rib and the iliac crest) over light clothing without any pressure to the body surface. Samples from peripheral blood were collected following 12 hours overnight fasting. Total cholesterol, HDL, LDL (low density lipoprotein cholesterol), TG, and fasting glucose (FBS) were measured by the enzymatic colorimetric method (using CHOD-PAP, POD-catalase , GPO-PAP and GOD-PAP methods) using commercial enzymatic reagents purchased from Pars Azmoon, Tehran, Iran. The instrument used for the analysis was Hitachi 704 autoanalyzer (Hitachi, Tokyo, Japan). Fasting serum insulin was detected in serum by an electrochemiluminescence method using Elecsys 2010 system (insulin Elecsys; Roche diagnostics, Boehringer mannheim, Germany). Blood pressure was measured with a validated mercury sphygmomanometer after the patient had taken a 15 minutes rest by sitting. Additional information regarding the age, medical history, smoking habits and current use of medications was obtained from the questionnaires completed during the screening. The MS severity was calculated as the average number of MS components present.

### 3.6. Statistical Methods

 For obtaining descriptive statistics, mean ± standard deviation (SD) or No. (%) were calculated, as appropriate. The χ2 test and Fisher’s exact test were used for categorical data. Student t-test and Mann-Whitney U-test were used for the analysis of continuous variables. Spearman correlation analysis was performed to correlate continuous variables. P values of < 0.05 was considered significant. SPSS version 18 was used for data analysis.

## 4. Results

**Figure 1.**
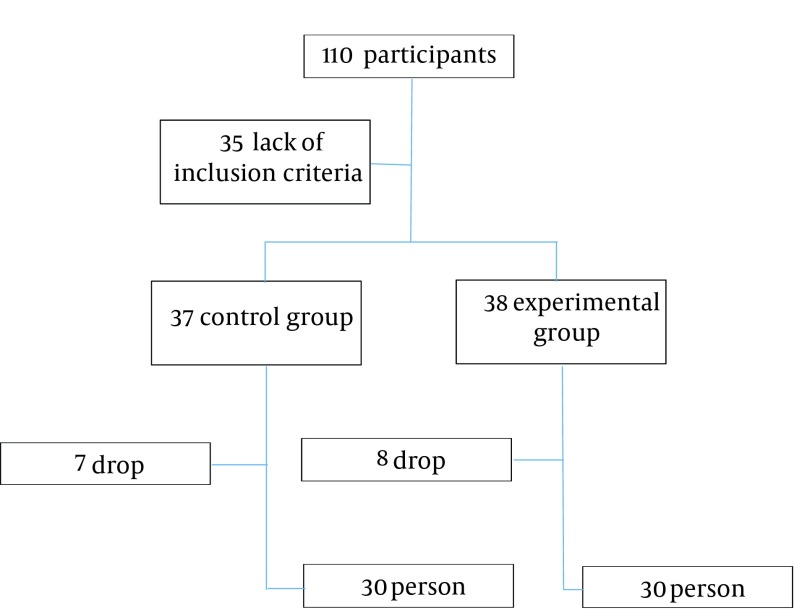
Significant Differencein the Severity of Metabolic Syndrome

Seven subjects from the control group and eight from experimental group dropped out before starting the diet. Results were collected and analyzed after follow up for two months. We did not find any harm or intolerance in both groups. The participants’ mean age was 45.41 ± 10.87 (SD) years. Mean BMI was 32.99 ± 5.01 (SD) kg/m^2^ (30.83 ± 5.82 (SD) kg/m^2^ in men and 33.36 ± 4.83 (SD) kg/m2 in women). Both groups were well balanced regarding the demographic characteristics, MS features and insulin (fasting) (Table 2). Two months after the intervention, the average change in any of MS components, BMI and weight were compared with the baseline in each group (Table 3). Significant (P < 0.001) decrease in waist circumference, weight and BMI were seen among those on the Razavi nutritional pattern. In this group, there were reductions in systolic and diastolic BPs, FBS and TG. However differences were not statistically significant. Cholesterol reduction was seen in both groups. Insulin reduction was non-significantly higher in the control group. The net reductions in waist circumference (-2.85), weight (-1.44) and BMI (-0.58) were significantly (P < 0.001) higher in the experimental group. Significant (P < 0.032) difference in metabolic syndrome severity was observed at the end of the study (Figure 1). 

**Table 2.** Comparison of Baseline Characteristics ^a, b, c^

Variables	Groups Experimental, n = 30	Control, n = 31	P Value
**Age**	46.11 ± 10.31	44.70 ± 11.55	0.639
**Waist**	105.17 ± 11.75	103.52 ± 11.80	0.586
**Weight**	83.63 ± 14.64	79.77 ± 12.65	0.275
**SBP**	140.43 ± 19.61	139.10 ± 17.68	0.781
**DBP**	89.00 ± 8.77	87.35 ± 6.80	0.415
**Chol**	201.63 ± 42.93	220.90 ± 53.60	0.127
**HDL**	41.10 ± 7.70	42.42 ± 6.88	0.483
**LDL**	125.50 ± 31.16	131.55 ± 44.50	0.542
**TG**	226.83 ± 119.37	206.10 ± 143.19	0.542
**FBS**	123.63 ± 59.63	115.94 ± 38.33	0.550
**Insulin**	8.40 ± 6.93	7.59 ± 8.91	0.697
**BMI**	33.38 ± 5.47	32.61 ± 4.59	0.552
**Metabolic syndrome severity**	0.61 ± 0.18	0.53 ± 0.25	0.139
**Gender, No. (%) ** ^**d**^			0.731
Female	25 (83.3)	27 (87.1)	
Male	5 (16.7)	4 (12.9)	
**Marriage, No. (%) ** ^**d**^			< 0.999
Married	24 (80.0)	25 (80.6)	
Single	6 (20.0)	6 (19.4)	
**Education, No. (%) ** ^**d**^			< 0.999
Lower Diploma	24 (80.0)	24 (77.4)	
Upper Diploma	6 (20.0)	7 (22.6)	

^a^ Abbreviations: BMI, body mass index; DBP, diastolic blood pressure; fasting glucose, FBS; HDL, high density lipoprotein cholesterol; LDL, low density lipoprotein cholesterol; SBP, systolic blood pressure; TG, triglyceride.

^b^ Continuous variables analyzed using independent t-test or Mann-Whitney test.

^c^ Data are presented as mean ± SD.

^d^ Categorical variables analyzed using Chi-square test.

**Table 3.** Comparison of Variables^a^

Variables	Time		Difference	P Value ^b^	Difference, 95% CI	P Value ^c^
	Before	After				
**Weight**						0.004
Experimental	83.70 ± 14.89	82.26 ± 15.67	-1.44	< 0.001	-1.18	
Control	79.77 ± 12.65	79.52 ± 12.70	-0.25	0.385	(-2.05,-0.32)	
**Waist**						< 0.001
Experimental	105.3811.90±	102.64 ± 11.80	-2.74	< 0.001	-3.38	
Control	103.42 ± 11.60	104.06 ± 11.14	0.64	0.330	(-4.80,-1.96)	
**SBP**						0.251
Experimental	140.62 ± 19.93	140.83 ± 17.66	0.21	0.469	-1.79	
Control	139.10 ± 17.68	141.10 ± 19.66	2	0.327	(-8.88,5.29)	
**DBP**						0.053
Experimental	88.97 ± 8.92	86.24 ± 8.57	-2.73	0.066	-4.14	
Control	87.35 ± 6.80	88.77 ± 8.86	1.42	0.273	(-7.85,0.42)	
**Cholesterol**						0.480
Experimental	201.79 ± 43.68	186.34 ± 28.61	-15.45	0.035	23.39	
Control	238.97 ± 108.99	200.13 ± 50.75	-38.84	0.002	(-12.71,59.49)	
**HDL**						0.986
Experimental	41.07 ± 7.84	42.72 ± 8.61	1.65	0.087	1.14	
Control	43.39 ± 9.35	43.90 ± 8.45	0.51	0.220	(-2.33,4.61)	
**LDL**						0.317
Experimental	125.55 ± 31.71	108.17 ± 26.79	-17.38	< 0.001	-4.21	
Control	131.55 ± 44.50	118.39 ± 41.68	-13.16	0.013	(-17.44,9.00)	
**TG**						0.119
Experimental	229.38 ± 120.65	174.00 ± 60.88	-55.38	< 0.001	-40.48	
Control	206.10 ± 143.19	191.19 ± 127.52	-14.91	0.258	(-84.04,3.09)	
**FBS**						0.126
Experimental	124.79 ± 60.34	114.59 ± 39.71	-10.2	0.125	-17.03	
Control	115.94 ± 38.33	123.03 ± 53.80	7.09	0.116	(-32.49,-2.11)	
**Insulin**						0.159
Experimental	12.07 ± 18.74	10.77 ± 20.26	-1.3	0.119	-4.62	
Control	7.61 ± 8.91	11.08 ± 25.84	3.47	0.036	(-15.39,6.13)	
**BMI**						< 0.001
Experimental	33.325.55±	32.73 ± 5.78	-0.59	< 0.001	-0.48	
Control	32.61 ± 4.59	32.51 ± 4.56	-0.1	0.361	(-0.83,-0.13)	
**Severity**						0.032
Experimental	0.61 ± 0.18	0.55 ± 0.23	-0.06	0.039	-0.10	
Control	0.53 ± 0.25	0.57 ± 0.21	0.04	0.296	(-0.20,-0.004)	

^a^ Data are presented as mean ± SD.

^b^ P value based on the paired sample t-test.

^c^ P value based on the independent test or Mann-Whitney test.

## 5. Discussion

In this study, the participants were randomly divided into two groups and could not communicate with each other. The laboratory staffs were also unaware of the group assignments. This design wipes out many confounding factors leading to a good generalization. Inability to determine as to whether individual components of the diet can account for the changes observed or those occurred in metabolic risk factors, was a limitation of this study. We assume that observed protective effects were due to the sum of all dietary changes. Although evaluation of the effect of each factor was rendered difficult in multiple dietary interventions, the advantage of a whole diet approach in prevention of CVD has been appreciated (16). The net reductions in waist circumference, weight, BMI were significantly (P < 0.001) higher in the experimental group. As most subjects with the MS are obese, first line focus of any dietary treatment should be on weight reduction. This can lead to improvement in insulin sensitivity and beneficially affect all other abnormalities related to MS. Weight reduction is a powerful measure in treatment of MS (17). Visceral adiposity is independently associated with insulin resistance, lower HDL, elevated TG levels, lower LDL particles, aortic stiffness, calcification of coronary artery, and higher BP. Although both insulin resistance and central body fat are associated with the MS, intra-abdominal fat is independently associated with all MS components, suggesting the possibility of a pathophysiological link. Waist circumference and TG may best indicate insulin resistance and visceral adiposity in those with a fasting plasma glucose < 6.4 mmol/L (18). The most common symptom of metabolic syndrome, the abdominal obesity which is also called 'dysfunctional adipose tissue' marker, is crucially important in the clinical diagnosis (19). In this study, significant reductions in weight, waist circumference and BMI were observed.

Comparing this study with the study conducted by Esposito on Mediterranean-style diet (20), revealed that the mean body weight in patients following the Mediterranean-style diet decreased 4.0 kg after 2 years, but mean body weight in patients following the Razvi- style diet decreased 1.44 kg after 2 months. BMI decreased by 1.2 after 2 years in patients on the Mediterranean diet. This reduction in BMI was 0.58 in patients on the Razvi diet after 2 months. Waist circumference reduction in patients following the Mediterranean-style diet was 2 centimeters after 2 years which was not significant. Patients following the Razavi-style diet experienced a decrease of 2.85 centimeters in waist circumference after 2 months. Reductions in systolic and diastolic BPs, HDL, TG and fasting plasma insulin were not significant following both Razavi-style diet and Mediterranean-style diet. FBS reduction of 9.63 in Razvi-style diet was not statistically significant while that of 8 cm in the Mediterranean-style diet was significant. Longer-term studies could elaborate more significant effects of this diet on other components of the metabolic syndrome. In conclusion, Razavi-style diet can have positive effects on weight loss, reduction in waist circumference and BMI that can relieve insulin resistance and other components of the metabolic syndrome. Thus, Razavi-style diet can be beneficial in reducing the risk of cardiovascular disease and diabetes.
